# Analysis of Tobacco Price Elasticity in Albania Using Household Level Data

**DOI:** 10.3390/ijerph17020432

**Published:** 2020-01-09

**Authors:** Aida Gjika, Edvin Zhllima, Klodjan Rama, Drini Imami

**Affiliations:** 1 Faculty of Economics, University of Tirana, Development Solutions Associates and CERGE-EI, 1001 Tirana, Albania; aida.gjika@gmail.com; 2 Faculty of Economics and Agribusiness, Agricultural University of Tirana, Development Solutions Associates and CERGE-EI, 1029 Tirana, Albania; dimami@ubt.edu.al; 3 Faculty of Agriculture and Environment, Agricultural University of Tirana and Development Solutions Associates, 1029 Tirana, Albania; krama@ubt.edu.al

**Keywords:** tobacco consumption, price elasticity, public health, Western Balkans, Albania

## Abstract

This paper analyzes the determinant factors of tobacco consumption in Albania, which is one of the countries with the highest smoking prevalence in Europe. To empirically estimate the elasticity of cigarettes demand in Albania, the paper uses the Living Standard Measurement Survey (LSMS) applying Deaton’s (1988) demand model. This paper estimates an Almost Ideal Demand System (AIDS), which allows disentangling quality choice from exogenous price variations using unit values from cigarette consumption. Following Deaton’s model, the results suggest that the demand for tobacco is inelastic, with a price elasticity of −0.57. The price elasticity appears to be within the range of elasticity estimates frequently reported for low- and middle-income countries. The results suggest that total expenditure, household size, male-to-female ratio, and adult ratio are important determinants of tobacco demand in Albania. The increase in the tobacco price, which has been mainly driven by increased excises, has demonstrated a significant impact on reducing tobacco consumption. Consequently, the Albanian government may engage in gradual increases in excise taxes given the inelastic tobacco demand.

## 1. Introduction

Smoking is known to cause many serious diseases and other health-related problems [1]. According to the World Health Organization, there are 1.1 billion smokers worldwide, of which about 7 million die each year from tobacco use. This figure is projected to grow to 8 million by 2030, with the vast majority (80%) of deaths anticipated to occur in low- and middle-income countries [2]. In addition to health problems, smoking incurs considerable economic costs not only to individual households but also to society as a whole. Goodchild and colleagues [3] estimated that the total economic cost of smoking, including both health expenditures and productivity losses, amounted to PPP $1852 billion (US$1436 billion) in 2012, equivalent to 1.8% of the world’s annual Gross Domestic Product (GDP)—almost 40% of this cost occurred in developing countries. As a consequence, there has been a growing awareness among policymakers to address the serious issue of smoking, especially in more developed countries.

Efficient policies aimed at reducing smoking prevalence need to be based on solid evidence. Tax policies on tobacco products are considered most efficient not only for reducing tobacco consumption but also for their positive impact on budget revenues, given the large sales volumes, the relatively inelastic demand, the lack of close substitutes, and the relative ease of administration and enforcement [4,5]. In terms of reducing tobacco consumption, both economic theory and empirical studies suggest that especially young people and people with low incomes or from low socio-economic status are more sensitive to tax and price increases, because the greater the share of an individual’s disposable income spent on a good, the more the individual will respond to price changes [6,7].

The positive impact of taxes on reducing tobacco consumption has been confirmed in several empirical studies on developed and developing countries [7,8,9,10,11]. Eozenou and Fishburn [8] argue that an increase in the price of cigarettes, irrespective of the level of income, would both prevent individuals from starting the use of tobacco and cause smokers to quit smoking. At the same time, the increase in price would have a positive effect on fiscal revenues, which in turn are closely linked to the demand elasticity. In developing countries, the evidence of consumer responses to price increases differs between countries. A review of studies estimating price elasticity for tobacco has shown there is a diversity of evidence reflecting whether the elasticity is higher [9] or lower in developing countries [10,11] compared to developed countries. This contrast supports the view that price elasticity may be country specific e.g., due to the market structure, patterns of consumption, and government role and associated policies [12,13,14,15,16]. For instance, some authors of studies in developing countries, Kenkel et al. [17], Lance et al. [18], and Liu et al. [10] find weak evidence of tax or price effect on tobacco demand. In addition to these studies Ross et al. [19] found that this trend might derive from the wide range of cigarette prices available on the market, high social acceptability of smoking, and stockpiling effects. Given the lack of clear outcomes from the literature, it is therefore important to provide results in a specific country or region. Research on the price responsiveness for tobacco products in Central and Eastern Europe, and particularly Western Balkan countries, is scarce. Most studies have focused on the institutional aspects of the enforcement of tobacco control policies, illicit trade, or quantities of tobacco consumption, with little regard to consumers’ responsiveness to price changes [19,20,21]. This paper, therefore, attempts to fill this gap by estimating the price elasticity of demand for cigarettes in Albania, using reported data on tobacco use at household level derived from the 2012 Living Standards Measurement Survey (LSMS).

Albania is an interesting case for exploring the elasticity of demand and searching for excise tax effects on cigarette demand, given the high and increasing consumption rate in previous years accompanied with a slight decrease in recent years. Moreover, although the tax rates have been increasing over the years, they are still among the lowest in Europe. Albania applies only the specific excise tax and the value added tax (VAT), with the excise tax on cigarettes at 48% of the sales price or 44.7 Euro per 1000 cigarettes. Meanwhile, the total tax burden on cigarettes (adding to excise tax the tariff on imports and VAT) for 2017 amounted to about 66.6% of the total price. The other countries in the region have made considerable progress in increasing cigarettes taxes to the benchmark level of 70% set forth in the Framework Convention for Tobacco Control (FCTC). For instance, according to the WHO’s recent report on Global Tobacco Epidemic, Montenegro and North Macedonia have moved to the top group for taxes comprising at least 75% of the retail price [22].

The paper is structured as follows. Section 2 describes and explains the materials and methods used to estimate the price elasticity of cigarette demand. Section 3 presents the estimation results and discusses their statistical and theoretical significance. Finally, Section 4 provides concluding remarks.

## 2. Materials and Methods

### 2.1. Country Bacground

Albania is a Western Balkans country with one of the highest smoking prevalence rates in the region [2]. According to WHO figures reported for 2015, smoking prevalence as a share of the adult population is 29.2%, and is dominated by males at 51.2% compared to 7.6% for females [2]. The yearly cost of tobacco consumption to the Albanian economy (including direct costs related to healthcare expenditures and indirect costs related to lost productivity due to early mortality and morbidity) are estimated around 270 million Euros. Lokshin and Beegle [23] estimated the negative effect of smoking on wages in Albania found that “every month the GDP of Albania is 2.6 percent lower compared with what it would be in the absence of smoking”. As far as household expenditures are concerned, the average yearly expenditure on cigarettes is over 10% of GDP per capita, or around $37 (these figures have been computed from the dataset of Albania´s Living Standard Measurement Survey (LSMS) of 2012). When compared with the average expenditure on cigarettes in 22 countries surveyed in the framework of the Global Adult Tobacco Survey (GATS) 2008–2013, these figures are relatively high. In 14 GATS countries, the average monthly expenditure on cigarettes is more than 5%, while only in Romania, Bangladesh, and Nigeria, it is over 10% [24]. These expenditure figures for Albania appear high despite the fact that the retail price of the most sold cigarette brand (MSB) in Albania is among the lowest in the region, about USD 2.2 per packet—North Macedonia has a lower average cigarette price [25]. The low cigarette price can be attributed to the low level of taxes on cigarettes.

The response of the Albanian government to this high incidence of cigarette consumption has increased with several policy measures implemented since 2000. In 2000, the government adopted a set of ‘National Tobacco Control Provisions’ which consisted of a ban on tobacco advertising on television, radio, printed media, and billboards; public information campaigns on the dangers of tobacco use; health warnings and the disclosure of tobacco product ingredients; and restrictions on smoking in public places such as educational and healthcare facilities, government buildings and public transport. The government signed in 2004 and ratified in 2006 the FCTC. According to the 2017 report of the World Health Organization, Albania ranks among the countries that have had the highest achievements in raising awareness and implementing anti-smoking legislation and tobacco advertising penalties [2]. As far as fiscal policies are concerned, the government has continuously raised taxes on tobacco products, even though at a much lower level as compared to other countries in the region [25].

Annual tobacco consumption in Albania is about 5000 tons with a deviation of plus or minus 10% in some years (http://open.data.al/en/lajme/lajm/id/1573/Prodhimi-dhe-konsumi-i-duhanit-dhe-cigares-2005-2014). Most tobacco products consumed in Albania are imported, given that Albania does not have a tobacco manufacturing industry. Data on tobacco consumption and expenditure are generally recorded by the Albanian Institute of Statistics (INSTAT) through the annual Household Budget Survey (HBS). To measure the changes in households´ living standards, INSTAT has also conducted four rounds of the LSMS in 2002, 2005, 2008, and 2012.

### 2.2. Data, Variables, and Model

The majority of studies estimating the price elasticity of demand are based on data collected from household surveys, such as the HBS. The HBS records budgetary expenses of respondent households, where the amounts spent on cigarettes and tobacco are part of the overall composition of household consumption. Similar to other studies (see for instance Giovino et al. [26]), our research relies also on household surveys.

In Albania, although monthly data on cigarette and tobacco consumption are available and derived mostly from import data, the quality and consistency of this data is low, due mainly to a share of uncontrolled tobacco produced by farmers which is largely self-consumed or sold illegally in the domestic market. Also, there are no prices at the regional level because the price data is reported only at the national level.

Given the limitations on data availability and consistency related to tobacco purchases in the HBS database, we rely on data from the 2012 LSMS, since it contains all the necessary information required to estimate the price elasticity of demand. We use the 2012 LSMS data because it is the most recent cross section survey. This survey is nationally representative with a sample of 6671 households covering both urban and rural areas across the 12 regions of Albania. The information used from the LSMS is from different modules such as Education, Poverty, Health, Purchases, and Labor. The Health module was the main focus because it contains information on cigarette consumption and the respective expenditures. Having data on our main variable of interest and other control variables at household level allows us to estimate the demand model, and consequently to estimate the elasticity of cigarettes demand by taking into account spatial variations in prices [27].

To estimate the elasticity of demand, using the LSMS data, we apply the methodology introduced by Deaton [27] and further developed by Deaton [28]. The Deaton model is a consumer behavior model that relies on household survey data and regional price differences. However, given the limited price information at the disaggregated level of segregation, as typically found in household surveys, unit values are used extensively as a proxy for prices. More precisely, as explained in Deaton [27], the unit value is the ratio of expenditure of a certain good over its quantity. While price data are rarely accurate and sufficient at household level [29], unit values are often considered superior to prices. However, prices and unit values are not identical. Measurement errors involving the quantity and variation in the quality of tobacco due to the heterogeneous nature of this product [30] (p. 9) are some of the reasons for the differences between prices and unit values. Though, it should be noted that the use of data based on statements by the representatives of households on the expenditure amount for cigarettes and quantity of use from a unit price have raised concerns in various studies [31,32]. Reliability of reporting is limited, since one member of a household is not able to assure the accuracy of the information on the quantity of cigarettes used by other members. In some cases, heaping—some respondents in surveys are likely to report rounded-off counts, particularly multiples of 20, 10, or 5; this is called ‘heaping’ and can bias the mean derived based on the respondent’s information—is likely to arise as a particular form of retrospective recall error. The use of self-declared data for both overall expenditures and tobacco expenditures might also create spurious correlation since the same biased estimation does influence both the dependent and the independent variables. Considering these facts, we used Deaton’s model and we are conscious about the gaps of this approach.

The Deaton model accounts only for positive quantities of tobacco, as such being limited only to the consumption decision. Indeed, there are other models, such as the two-part model [33] or double-hurdle approach, which simultaneously incorporate the participation and consumption decision. However, accounting for non-smokers, in addition to smokers, does not infer that these households have sheer abstention. A possible rationale might be either a budget constraint (corner solutions) to purchase this good or abstention. Whilst the denial of the latter constraint is often criticized, it should be noted that relying on the weak separability assumption might distort the results of the elasticity. Hence, this study relies on the weak separability assumption, which implies that smokers and non-smokers have different preferences for tobacco, which favors the use of conditional demand models (only for positive consumption of tobacco) by making it robust to other approaches [34]. Nevertheless, additional investigation is warranted in terms of a more comprehensive approach of the behaviors of consumers towards tobacco by acknowledging both the participation and intensity of smoking.

Deaton’s model using unit values consists of two equations

(1) $$w_{hc}=\;\alpha^{0}+\;\beta^{0}lnx_{hc}+\;\gamma^{0}\cdot z_{hc}+\;\theta lnp_{c}+\;u_{hc}^{0}$$

(2) $$lnv_{hc}=\;\alpha^{1}+\;\beta^{1}lnx_{hc}+\;\gamma^{1}\cdot z_{hc}+\;\varphi lnp_{c}+\;u_{hc}^{1}$$

where, subscripts *h* and *c* in Equations (1) and (2) represent the household and regional cluster, respectively. The dependent variable *w* in Equation (1), denotes the budget share of the household on cigarettes in percentages, whereas *v* in Equation (2) represents the unit values. The right-hand side variables *x*, *z*, *p* in both equations represent the total expenditures of households, other household characteristics and cigarette price, respectively. The remaining terms, $u_{hc}^{0}$ and $u_{hc}^{1}$ represent the error terms.

Regressing budget shares on real expenditures and prices, as displayed in Equation (1)*,* resemble the Almost Ideal Demand System (AIDS) of Deaton and Muellbauer [35]. However, following Deaton [28] (pp. 15–16), it should be noted that Equation (1) and AIDS are not the same for two reasons. First, Equation (1) (similarly also Equation (2)) should not be considered as a representation of preferences of consumers for goods, but purely as regressions of budget shares and unit values. Second, the AIDS regresses quantities on prices, which is different to the framework of Equation (1) where consumers choose both quantities and qualities of products, whereby expenditures for a certain good are a product of quantity, quality and price.

Following Deaton’s model feature of failing to observe prices for the goods [28] (p. 5) due to the assumption that prices are the same across households in the same cluster, it is possible to estimate consistent estimates of the remaining parameters (except *h*) by using cluster deviation-from-the-mean approach. Because prices are cluster-invariant, this approach removes the effect of prices from both equations. From a technical perspective, the estimates from the above approach are calculated by including dummy variables for each cluster.

An important note of caution is in order when referring to the unit value equation. The unit values (the share of household expenditure to quantity purchased) is calculated only for those households which have purchased in the tobacco market. Otherwise, there will be no unit values calculated. As previously mentioned, unit values represent the variation, due to the changes in prices of cigarettes not only in relation to the quantity but also to the quality of cigarettes (different brands of cigarettes). Accordingly, a consumer of cigarettes might either consume fewer cigarettes (quantity reduction), or consume the same amount of cigarettes but of a lower quality (quality shading), while assuming that the expenditures for cigarettes remain the same.

Hence, referring to Equation (2), the coefficient on the total expenditures ($\beta^{1}$) represents the expenditure elasticity of quality, different from the coefficient on price (ψ) that represent changes in the unit value of cigarettes due to changes in prices. In the absence of quality shading, the latter coefficient would be equal to one, whereas the former coefficient would be zero. On the other hand, the corresponding coefficient in Equation (1), more precisely the semi-elasticity of price θ, cannot be observed. Nevertheless, including prices in both Equations (1) and (2), the Deaton model enables the estimation of the θ parameter. Equation (2) is re-arranged so the logarithm of prices is moved to the left-side of the equation, whereas unit values, household total expenditure, other control variables for the household characteristics and error terms are placed on the right-side of the equations. Additional transformation up to a linear relationship between the budget share and unit values as well as other control variable would yield

(3) $$w_{hc}=\;\alpha^{2}+\;\beta^{2}lnx_{hc}+\;\gamma^{2}\cdot z_{hc}+\;\hat{\phi }lnv_{hc}+\;u_{c}^{2}$$

Following Deaton [28] (p. 19), the estimation of the model is in three stages. First, Equations (1) and (2) are estimated by using cluster means subtracted from the data (deviation from the mean approach). Second, the estimates from the first stage, while having removed the effect of total household expenditure and other control variables from the budget share and unit values, are used to estimate unit value elasticity of consumption. In the third stage, the effect of price elasticity is separated from the quality effects using the separability assumption (see Appendix A for the mathematical transformations). Finally, following John [34], the bootstrapping procedure (1000 replications) is used to determine whether the above elasticity is statistically significant.

## 3. Results

Before interpreting the results, a note of caution is in order when referring to the definition of clusters. Clusters are defined based on the information of the primary sampling units. In this empirical analysis, overall, there are 603 clusters defined. In term of the descriptive statistics of variables used in our model, there are several control variables used such as total expenditures in logarithmic form, household size in logarithmic form, gender and composition of households and urban/rural status. The descriptive statistics of all variables used in the estimation of the Deaton model are reported in Table 1. Before reporting the results, it is important to note that households with zero expenditure are not considered in the analysis, given the ‘fundamentally different’ preferences of cigarettes between consumer and the non-consumers [34]. The non-smoker households are not counted due to the provided argument and limited evidence in other studies [34] that tobacco is not part of their utility function and that tobacco price cannot directly influence it.

As shown in Table 1, the households that are considered in our model have an average male ratio of about 54%. On the other hand, mean and maximum years of education of 10.5 and 12.2 suggest that on average adult household members have a secondary level of education. Furthermore, about 50% of the households are from urban areas.

Using the Deaton model, the estimated results at household level are presented in Table 2. Starting with the results of the unit values equation, it is important to note that the coefficient for total expenditure, which is statistically significant, represents the quality elasticity of expenditure. More precisely, this elasticity is about 0.5%, which suggests that households with 10% higher expenditure will buy cigarettes that are about 5% more expensive.

Moving to the other control variables, the results suggest that the unit value is lower in larger households, and in households with more men and elderly. Surprisingly, the results suggest that there is no statistical effect of education (neither mean nor maximum years of education) on the unit value. A note of caution is required when referring to the rural settlements dummy variable, which appears to be omitted from the regression. Next, attention is devoted to the cluster fixed effects, which are statistically significant, indicating that spatial variation among clusters is important and needs to be considered in the analysis.

Moving to the estimated coefficients from the budget share equation, it is noted that households with a higher level of expenditure spend a lower share of their budget on cigarettes. More precisely, the coefficients on total expenditure suggest that households with 10% higher expenditure spend about 0.2 percentage points less of their budget on cigarettes. Also, budget shares on cigarettes are lower among larger households, and with higher mean years of education, but larger in households with more males. Similar to the unit value regression results, the cluster fixed effect remains significant, highlighting the importance of spatial variation.

Exploring further the regional effects from the variability of unit values and budget share, the results emphasize once more the importance of taking into account the regional differences. For instance, households in Tiranë and Durrës regions—the largest and most developed regions in Albania—pay higher prices compared to the base category (Berat region), which is a midsize and average-developed region. More specifically, the households in the base category spend about 8.8% of their budget on cigarettes, while households in the other regions spend on average from 1.2 percentage points more to 3 percentage points less of their budgets on cigarettes. Following the subsequent stages of the Deaton model, by removing the effect of total household expenditure and other control variables from the budget share and unit values, and additionally purging regional effects from the budget share and unit values variability, the results suggest the importance of regional preferences in both budget share and unit values in terms of cigarettes, as observed by the significance of regional effects (Cluster Dummies) reported in Table 2.

At the final stage, the price elasticity is separated from the quality effects resulting in a distinct elasticity of total expenditure, also known as the quality elasticity or income elasticity. Hence, we obtain the price elasticity of cigarette demand of −0.57 and an income elasticity of 0.24. Namely, if cigarette prices would increase in Albania by 10%, the quantity demanded for cigarettes would decrease by 5.7%. Similar results were obtained by following the bootstrapping procedure. The results suggest that the demand for cigarettes is inelastic (ξ = −0.551; SEξ = 0.094, t = 5.861, thus statistically significant). However, the estimated price and income elasticity should be interpreted with caution due to data quality of the LSMS. Despite continuous improvement over years in conducting the LSMS survey, which meant the latest 2012 LSMS was used for this study, consistent improvement of data collection and recording is required, especially in terms of reliability of data. Also, a note of caution is required when referring to this elasticity as it only indicates the elasticity at the intensive margin, namely for households with positive cigarettes consumption.

In this context, an increase of income by 10% is associated with a 2.4% increase in quantity demanded, all else equal. Considering the above price elasticities of demand and income, the impact of excise tax increases on budget revenues would be considerable. In 2017, total budget revenues generated from tobacco taxation amounted to around 400 million Euros, where the share of revenue to GDP is 1.4%, and the share of tobacco revenue in relation to the total budget revenue is 5.1%. The largest bulk of the revenue comes from the excise tax. Taking 2017 as a reference year and assuming a similar fiscal performance, our calculations show that the budget revenues from increasing the excise tax would increase by 40 million Euros or 33.7%. This means that revenues from total tax would increase by 43.4 million Euros (increase of 26.4%), including also the positive effect on VAT of 8.7%.

## 4. Discussion and Conclusions

Tobacco consumption in Albania remains a pressing health and economic issue. Although several institutional changes to tobacco control policies have been undertaken during recent years, their enforcement is continuously challenged by the strength of institutions and the success of fiscal reforms. Being a typical developing and transition economy, it is expected that its revenue system will continue to rely on the categories of indirect taxes, such as tobacco excises. Thus, the policy approach for controlling tobacco consumption in Albania involves both fiscal and non-fiscal instruments.

The analysis of the consumers’ responsiveness to price changes showed an elasticity of demand for cigarettes of −0.57. This level of elasticity suggests that increasing the tax burden on cigarettes would have a significant positive impact on the reduction in cigarette demand. If prices would increase by 10%, the demand for cigarettes would fall by 5.7%. The empirical results converge with the range of elasticity coefficients found in low- and middle-income countries (from −0.50 to −1.00 [9]), yet differ from other countries such as Ukraine [11]. Albania has a relatively narrow range of cigarette prices in the market (share of most expensive to least expensive is 1:2), weak stockpiling effects, and relatively low social acceptability of smoking in urban areas.

The country’s consumption patterns and model observations reflect a domination of adult male consumers, coming from larger households. When considering households’ income changes, the average level of education is negatively related to tobacco consumption. Also, spatial differences across the country are important and need to be taken into account.

In addition to the type of country, indeed, price elasticity may differ in long-run vs. short-run. John [34] argues that long-run elasticity coefficients appear to be relatively higher than for the short-run. It should be noted that this paper is that it does not assess long term elasticity, since the data analysis is cross-section. Future research should consider panel data or other forms of time series analysis to assess long-run elasticity.

The results of the study are subject to several limitations. The study does not take into account the overall preferences for cigarettes since it does not include households with zero expenditure. Whilst the empirical literature on the relative impact of prices on intensive and extensive margins appear to be somewhat mixed [36], and mostly arguing on only statistical significance of accounting zero-consumption households [34], this study focused on the intensive elasticity, while acknowledging a theoretical effect of price on the initiation of smoking. We note also the differences of these results compared with the real elasticity of smoking which incorporates propensity of people to quit smoking as a result of price increase. A further analysis could take account also of the rational addictive behavior of smokers such as Becker–Murphy model [37], which accounts for the tolerance, reinforcement, and withdrawal characteristic of addictive consumption.

The figures on overall cigarette tax burden as well as the budgetary trends provide optimal conditions for strengthening fiscal control instruments on tobacco in Albania. Considering the current level of tax burden on cigarettes and other tobacco products is the lowest in the region, there is room for increasing taxes, especially excise taxes. That will not only bring about health-related positive impacts, but also generate additional budget revenues.

The potential positive fiscal effects on tobacco reduction and budget revenues could be affected by the inability of the tax administration system to control tobacco production, imports and sales [11,20,21]. Therefore, the process of increasing the excise tax should be undertaken jointly with serious efforts for improving administrative capacities for tax collection. The study results are subject to several limitations. A part of it derives from the use of household stated expenditures of household representatives. First, the data do not allow controlling for heaping. Moreover the estimations do not capture the ‘downtrading’ dimensions (some cigarette smokers switching or ‘downtrading’ to lower priced cigarette brands or to hand-rolling tobacco (HRT), as a reaction to increased cigarettes prices due to excise increase). Furthermore, the lack of data availability on the age and gender of tobacco users, due to lack of individual data, does not allow us to estimate price elasticity by age or gender. Elasticity coefficients differ in terms of age, gender and incomes, time observed, and brands [10,38]. The youth in several low-income countries is found to be more responsive to cigarette price changes than other age cohorts [39,40]. As a result, it becomes difficult in providing policymakers evidences for possible use of tailored fiscal and non-fiscal policies.

Despite the limitations, the results provide strong evidence for supporting an increase in the excise tax for tobacco having a direct effect on smoking. Moreover, immediate measures for pursuing sustained incremental increases in tobacco excises are feasible and in line with the revenue-raising objectives of the governments. In order to avoid possible distortions, such as downtrading and stockpiling, the process of excise increase should be based on an ex-ante evaluation of the expected effects on demand, a rigorous application in terms of time of the excise schedule as well as strict monitoring (ex-post) of the impact on price, quantity, and tax revenue.

In addition to the positive effect on overall budget revenues, raising tobacco excises can have also an income distributional impact on the population, given the fact that tobacco excise increases are likely to be progressive [41]. The main target group that could significantly benefit from the reduced tobacco consumption and from increased budget revenues could be the poorest people. In a study on the impact on health and other distributional consequences of increasing the excise tax, Salti et al. [42] found that savings accrued from reduction in health spending would be associated with decreased income rates especially among the poorest quintile of population. This is relevant also for Albania given the significant regional variation in household expenditures on tobacco products and given that the highest share of these expenditures is incurred by households in the poorest regions.

## Figures and Tables

**Table 1:** Descriptive statistics of variables

	Mean	Std dev	Min	Max
Unit Value, Cigarettes (ln)	4.02	0.78	0.15	6.96
Budget share, Cigarettes	0.08	0.05	0.001	0.44
Total expenditure (ln)	13.12	0.39	11.70	14.66
Household size (ln)	1.35	0.42	0	2.77
Male ratio	0.54	0.16	0	1
Adult ratio	0.84	0.19	0.22	1
Mean education (years)	10.51	1.92	4.25	17.62
Maximum education (years)	12.22	2.73	8.5	20
Rural Settlements (dummy)	0.50	0.50	0	1
Number of observations	1551			

Author’s calculation based on the LSMS data. Note: Conditional on being in the Deaton Model.

**Table 2:** Regression results.

Variables	Unit Value (per pack, ln)		Cigarettes Budget Share (in %)	
Total expenditure (ln)	0.525 ***	(0.057)	−0.018 ***	(0.004)
Household size (ln)	−0.270 ***	(0.053)	−0.012 **	(0.003)
Male ratio	−0.249 **	(0.101)	0.019 **	(0.007)
Adult ratio	−0.205 **	(0.099)	−0.001	(0.007)
Mean education	−0.006	(0.017)	−0.002 *	(0.001)
Maximum education	0.013	(0.011)	−0.001	(0.000)
Rural Settlements	Omitted			
Cluster dummies	F(602, 943)		F(614, 1061)	
	4.266 ***		4.218 ***	
Constant	−2.299 ***	(0.701)	0.339 ***	(0.051)
Observations	1543		1682	
R-squared	0.764		0.720	

Standard errors in parentheses *** *p* < 0.01, ** *p* < 0.05, * *p* < 0.1. Author’s calculation based on the LSMS data.

## Appendix A. Mathematical Derivation

Proceeding with the Deaton’s model with unit values, this model consists of two equations

(A1) $$w_{hc}=\;\alpha^{0}+\;\beta^{0}lnx_{hc}+\;\gamma^{0}\cdot z_{hc}+\;\theta lnp_{c}+\;u_{hc}^{0}$$

(A2) $$lnv_{hc}=\;\alpha^{1}+\;\beta^{1}lnx_{hc}+\;\gamma^{1}\cdot z_{hc}+\;\varphi lnp_{c}+\;u_{hc}^{1}$$

where, subscripts *h* and *c* both on Equations (A1) and (A2) represent the household and regional cluster, respectively. The dependent variable w in Equation (A1), denotes the budget share of household on cigarettes in percentages, whereas v in Equation (A2) represents the unit values. The right-hand side variables on both equations *x*, *z*, *p* represent the total expenditures of households, other household characteristics and cigarette price, respectively. The remaining terms, $u_{hc}^{0}$ and $u_{hc}^{1}$ represent the error terms.

Additional transformation up to a linear relationship between the budget share and unit values, accounting as well the other control variable, would yield Equation (A3):

(A3) $$w_{hc}=\;\alpha^{2}+\;\beta^{2}lnx_{hc}+\;\gamma^{2}\cdot z_{hc}+\;\hat{\phi }lnv_{hc}+\;u_{c}^{2}$$

As introduced in Section 2.2, the estimation of the model, according to Deaton [28], goes under three stages. After estimating Equations (1) and (2) by using cluster means subtracted from the data, the estimates from the first stage, would be

(A4) $${\tilde{y}}_{hc}^{0}=\;w_{hc}-\;{\tilde{\beta }}^{0}lnx_{hc}-\;{\tilde{y}}^{0}z_{hc}$$

(A5) $${\tilde{y}}_{hc}^{1}=\;lnv_{hc}-\;{\tilde{\beta }}^{1}lnx_{hc}-\;{\tilde{y}}^{1}z_{hc}$$

Second, creating cluster averages of budget shares and unit values (as displayed in Equations (A6) and (A7)) and using the estimated variance and covariance from estimated residuals in Equations (A1) and (A2), the parameter ϕ from Equation (A3) is calculated as

(A6) $$\hat{\phi }=\;\frac{cov\left({\tilde{y}}^{0},\;\;{\tilde{y}}_{c}^{1}\right)-\;{\tilde{\sigma }}^{01}/n_{c}}{var\;\left({\tilde{y}}_{c}^{1}\right)-\;{\tilde{\sigma }}^{11}/n_{c}^{+}}$$

In the third stage, recalling the unit value definition and the assumption of weak separability, the parameter *θ* is calculated as

(A7) $$\theta =\phi /\left[1+\left(w-\;\phi \right)\frac{\beta^{1}}{\beta^{0}+w\left(1-\beta^{1}\right)}\right]$$

Based on Deaton [28] and John [30], if the estimated unit value elasticity of expenditure is close to zero, the quality shading and price semi-elasticity will yield an unbiased estimate of the parameter ϕ. However, in the presence of quality shading, the *θ* parameter needs to be corrected for bias (more precisely needs to be corrected downwards). Accordingly, the final formula for the elasticity will be

(A8) $$\epsilon_{p}=\left(\frac{\theta }{w}\right)-\;\varphi$$
